# Sleep-Promoting Effects and Possible Mechanisms of Action Associated with a Standardized Rice Bran Supplement

**DOI:** 10.3390/nu9050512

**Published:** 2017-05-18

**Authors:** Hyejin Yang, Minseok Yoon, Min Young Um, Jaekwang Lee, Jonghoon Jung, Changho Lee, Yun-Tai Kim, Sangoh Kwon, Boknam Kim, Suengmok Cho

**Affiliations:** 1 Division of Functional Food Research, Korea Food Research Institute, Seongnam 13539, Korea; Yang.Hye-jin@kfri.re.kr (H.Y.); msyoon@kfri.re.kr (M.Y.); myum@kfri.re.kr (M.Y.U.); jklee@kfri.re.kr (J.L.); jjh03201012@gmail.com (J.J.); chang@kfri.re.kr (C.L.); ytkim@kfri.re.kr (Y.-T.K.); 2 S&D Research and Development Institute, Osong 28156, Korea; so-kwon0004@hanmail.net; 3 Department of tourism Food Service Cuisine, Hallym Polytechinic University, Chuncheon 24210, Korea; bnkim@hsc.ac.kr

**Keywords:** rice bran, hypnotic, sleep-promoting, caffeine-induced arousal, histaminergic

## Abstract

Natural sleep aids are becoming more popular due to the widespread occurrence of sleep disorders. The objective of this study was to assess the sleep-promoting effects of rice bran—a product that is considered as a functional ingredient. To evaluate the sleep-promoting effects of a standardized rice bran supplement (RBS), we employed a pentobarbital-induced sleep test and conducted analyses of sleep architecture. In addition, the effect of RBS on a caffeine-induced sleep disturbance was investigated. Oral administration of RBS (500 and 1000 mg/kg) produced a significant decrease in sleep latency and increase in sleep duration in pentobarbital-induced sleep in mice. Moreover, both RBS (1000 mg/kg) and doxepin hydrochloride (histamine H_1_ receptor antagonist, 30 mg/kg) counteracted a caffeine-induced sleep disturbance in mice. In terms of sleep phases, RBS (500 mg/kg) promoted non-rapid eye movement sleep for the first 3 h following its administration. Lastly, we unveiled a possible mechanism for RBS action as the hypnotic effect of RBS was blocked by a histamine H_1_ receptor agonist. The present study revealed sleep-promoting effects of RBS using various animal assays. Such effects seem to be mediated through the histaminergic system. Our findings suggest that RBS may be a promising natural aid for relieving sleep problems.

## 1. Introduction

Sleep, which accounts for one-third of the human lifespan, plays a critical role in maintaining health and well-being [1]. The quantity and quality of sleep greatly contributes to physical and cognitive performance, immune system functioning, mood stability, productivity, and quality of life [2]. Although sleep has a significant impact on health and quality of life [3], insomnia is a current widespread health complaint, and has become a prevalent and disruptive problem in modern industrial societies [4,5]. Insomnia is generally characterized by difficulty initiating or maintaining sleep, resulting in non-restorative sleep and impaired daytime functioning [6]. According to numerous surveys conducted worldwide, 10–15% of the adult population suffers from chronic insomnia, while an additional 25–35% has transient or occasional insomnia [5].

Sedative-hypnotics, such as GABA_A_-benzodiazepine (BZD) receptor agonists and histamine H_1_ receptor (H_1_R) antagonists, are commonly used to treat insomnia. However, such hypnotics may objectively decrease sleep quality and are associated with side-effects that range from impaired cognitive function and memory to disruptions in general daytime performance [7,8]. To avoid such side effects, interest in the use of complementary and alternative medicine for insomnia has grown over the past two decades [9,10]. In particular, natural or herbal sleep aids are gaining gradual global acceptance due to their safety and efficiency [11]. A variety of dietary and herbal supplements, including valerian (*Valeriana officinalis*), hops (*Humulus lupulus*), 5-hydroxytryptophan, and suanzaoren (*Ziziphus jujuba*), are readily available. In addition, many researchers still focus on the sleep-promoting effects of medicinal plants and their constituents.

Rice bran is a by-product of the rice milling process and has a variety of merits, such as large production quantity, low cost, convenience of collection, as well as therapeutic and nutrition potential. In particular, rice bran is considered a promising source for dietary supplements and nutraceuticals [12,13,14]. Despite a wide spectrum of biological activities, studies investigating the sleep-promoting effects of rice bran have not been conducted. Therefore, in the present study, we assessed the effects of a rice bran supplement (RBS) on sleep and explored its possible mechanisms of action. For the evaluation of hypnotic effects, we employed a pentobarbital-induced sleep test along with electroencephalogram (EEG) and electromyogram (EMG) to analyze sleep architecture. In addition, the effect of RBS on caffeine-induced wakefulness (Wake) was tested.

## 2. Materials and Methods 

### 2.1. Materials

A standardized rice bran supplement (RBS, lot No. SD-RB-002) was obtained from S and D Co., Ltd. (Cheongju, Korea). According to the manufacturer, rice (*Oryza sativa* L.) bran was extracted with ethanol/water solution at 40 °C for 8 h. Extraction solutions were then filtered and concentrated. Finally, the concentrated solution, mixed with d-α-tocopherol, polysorbate 20, sodium caseinate, and dextrin, was dried and powdered. The yield of RBS was 29.3% (g RBS/g rice bran) and the proximate composition of RBS is shown in Table 1. RBS was standardized to contain 4.5 mg/g of γ-oryzanol. Pentobarbital and caffeine (product number: C0750) were purchased from Hanlim Pharm. Co. Ltd. (Seoul, Korea) and Sigma-Aldrich Inc. (St. Louis, MO, USA), respectively. Histamine H_1_ receptor (H_1_R) antagonist doxepin hydrochloride (DOX) and H_1_R agonist 2-pyridylethylamine dihydrochloride (PEA) were purchased from Tocris Bioscience (Bristol, UK). The GABA_A_-BZD receptor agonist, diazepam (DZP), and antagonist, flumazenil (FLU), were purchased from Myungin Pharm. Co. Ltd. (Seoul, Korea) and Sigma-Aldrich Inc., respectively.

### 2.2. Animals

All procedures involving animals were conducted in accordance with the guidelines of the Korea Food Research Institutional Animal Care and Use Committee (permission number: KFRI-M-15018). Imprinting control region (ICR; male, 18–22 g, four weeks old) and C57BL/6N (male, 27–30 g, 12 weeks old) mice were purchased from Koatech Animal Inc. (Pyeongtaek, Korea). All animals were housed in an insulated, sound-proof recording room maintained at an ambient temperature of 23 ± 0.5 °C, with a constant relative humidity (55 ± 2%) on an automatically controlled 12-h light/dark cycle (lights off at 17:00). Mice had free access to food and water, and all efforts were made to minimize animal suffering and the number of animals required for the production of reliable scientific data.

### 2.3. Drug Administration 

All drugs were dissolved in sterile saline containing 5% tween 80 immediately before use, and administered by oral gavage to the mice using a sonde needle. In the mechanism investigation, FLU and PEA were injected intraperitoneally (i.p.) 10 min before drug administration.

### 2.4. Pentobarbital-Induced Sleep Test

**Figure 1:**
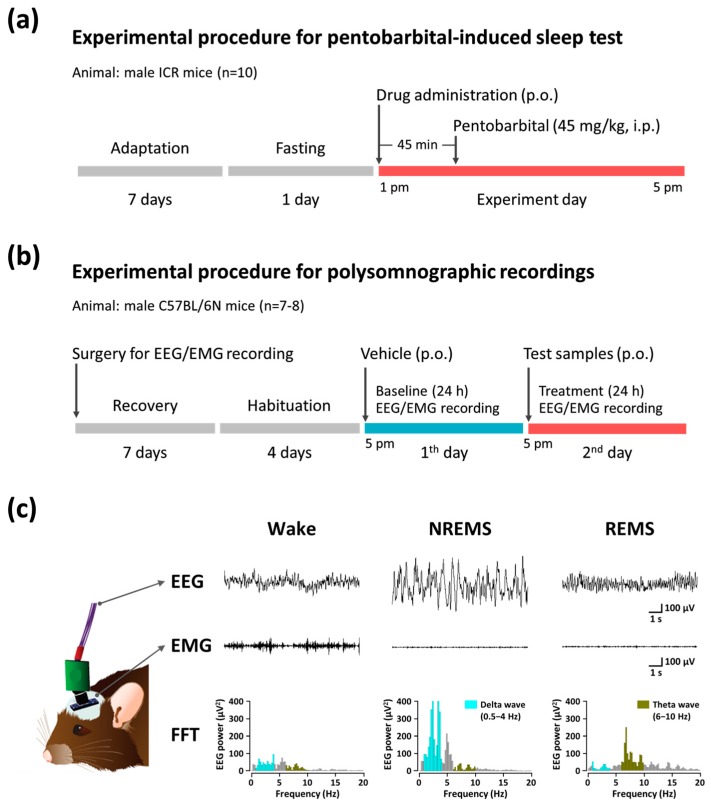
Experimental procedures and timelines for the pentobarbital-induced sleep test (**a**) and polysomnographic recordings (**b**,**c**) showing typical EEG and EMG waveforms, and FFT spectra in mice. EEG, electroencephalogram; EMG, electromyogram; FFT, fast Fourier transform; REMS, rapid eye movement sleep; NREMS, non-REMS; Wake, wakefulness.

The experimental procedure and timeline for the pentobarbital-induced sleep test is shown in Figure 1a. All experiments were performed between 1:00 p.m. and 5:00 p.m., and mice were fasted for 24 h before experiment onset. Mice (*n* = 10) orally received drugs 45 min before pentobarbital injection. Control mice (5% tween 80-saline, 10 mL/kg) were tested in parallel with animals receiving drug treatments. After the administration of pentobarbital (45 mg/kg, i.p.), mice were placed in individual cages and observed for measurements of sleep latency and duration. Sleep latency was recorded from the time of pentobarbital injection to the time of sleep onset, and sleep duration was defined as the difference in time between the loss and recovery of the righting reflex. Observers who scored these variables were blinded to the treatment groups.

### 2.5. Analysis of Sleep Architecture

The experimental procedure and timeline for sleep analysis is shown in Figure 1b. Under pentobarbital anesthesia (50 mg/kg, i.p.), C57BL/6N mice were chronically implanted with a head mount (#8201, Pinnacle Technology Inc., Lawrence, KS, USA) installed with EEG and EMG electrodes for polysomnographic recordings. The front edge of the head mount was placed 3.0 mm anterior to the bregma, and four electrode screws for EEG recording were positioned in holes perforated into the skull. Two EMG wire electrodes were inserted into the nuchal muscles. The head mount was fixed to the skull with dental cement. After surgery, mice were allowed to recover in individual cages for one week, after which they were habituated to the recording conditions for 3–4 days before the experiment. The EEG and EMG recordings were carried out by means of a slip ring design so that the movement of the mice was not restricted. EEG and EMG data were recorded using the PAL-8200 acquisition system (Pinnacle Technology Inc., Lawrence, KS, USA). Signals were amplified (100×), filtered (low-pass filter: 25 Hz EEG and 100 Hz EMG), and stored at a sampling rate of 200 Hz. Sleep states were monitored for a period of 48 h, which comprised baseline and experimental days. Baseline recordings were taken for each animal over the course of 24 h (beginning at 17:00 h), and served as controls for the same animal. Mice were considered asleep when no EMG signal was detectable. Vigilance states were automatically classified by a 10 s epoch as Wake, rapid eye movement sleep (REMS), or non-REM sleep (NREMS) by SleepSign ver. 3.0 (Kissei Comtec, Nagano, Japan). As a final step, defined sleep-wake stages were examined visually and corrected if necessary. Sleep latency was defined as the time from drug administration to the appearance of the first NREMS episode lasting for at least 120 s. Bouts of NREMS, REMS, and Wake were defined as periods of one or more consecutive epochs (each epoch: 10 s). Representative EEG/EMG waveforms and fast Fourier transform (FFT) spectrum of delta and theta waves are shown in Figure 1c.

### 2.6. Statistical Analysis

All data are expressed as the mean ± standard error of mean (SEM). Statistical analysis was performed with the Prism 5.0 (GraphPad Software Inc., San Diego, CA, USA). For multiple comparisons, data were analyzed using a one-way analysis of variance (ANOVA) followed by Dunnett’s test. Comparisons between two groups of data were analyzed by an unpaired Student’s *t*-test. The significance level was set at *p* < 0.05 for all statistical tests.

## 3. Results

### 3.1. RBS Potentiates Pentobarbital-Induced Sleep in Mice

**Figure 2:**
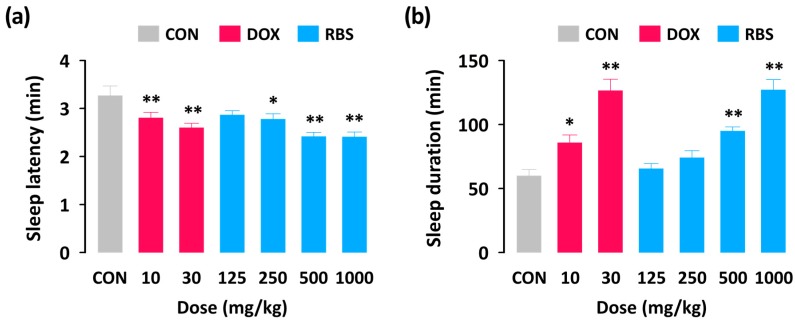
Effects of the administration of RBS or DOX on sleep latency (**a**) and sleep duration (**b**) in pentobarbital-treated (45 mg/kg, i.p.) ICR mice. Drugs were administered (p.o.) to mice 45 minutes before pentobarbital injection (i.p.). Each column represents mean ± SEM (*n* = 10). * *p* < 0.05, ** *p* < 0.01, significantly different when compared to the CON group (Dunnett’s test). CON, control; DOX, doxepin hydrochloride; RBS, rice bran supplement.

With a hypnotic dose of pentobarbital (45 mg/kg, i.p.), sleep latency and duration in the control group were 60.1 ± 4.7 and 3.3 ± 0.2 min, respectively (Figure 2). As expected, the positive control DOX (10 and 30 mg/kg, p.o.) significantly decreased sleep latency (Figure 2a) and increased sleep duration (Figure 2b) as compared to the control group. RBS (500 and 1000 mg/kg, p.o.) also caused a significant decrease in sleep latency and increase in sleep duration. The effects of RBS at 1000 mg/kg were similar to those of the positive control (DOX) at 30 mg/kg. This result suggests that, similar to DOX, RBS can act as a hypnotic agent to accelerate pentobarbital-induced sleep in mice.

### 3.2. RBS Attenuates a Caffeine-Induced Sleep Disturbance in Mice

**Figure 3:**
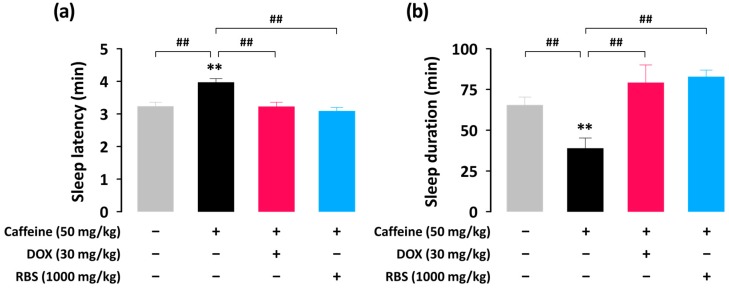
Effects of DOX (30 mg/kg) or RBS (1000 mg/kg) co-administration with caffeine (50 mg/kg) on sleep latency (**a**) and sleep duration (**b**) in pentobarbital-treated (45 mg/kg, i.p.) ICR mice. Drugs were administered (p.o.) to mice 45 min before pentobarbital injection (i.p.). Each column represents mean ± SEM (*n* = 10). ** *p* < 0.01, significantly different when compared to the CON group (Dunnett’s test). ## *p* < 0.01, significant difference between two groups. CON, control; DOX, doxepin hydrochloride; RBS, rice bran supplement.

In order to identify additional hypnotic effects of RBS, we investigated whether RBS could attenuate a sleep disturbance introduced by caffeine–a routinely consumed stimulant that may cause symptoms of insomnia [15,16]. In the present study, caffeine (50 mg/kg, p.o.) significantly (*p* < 0.01) inhibited pentobarbital-induced sleep in mice due to its well-known wake-promoting effect (Figure 3). Co-administration of RBS (1000 mg/kg) or DOX (30 mg/kg) with caffeine (50 mg/kg) significantly (*p* < 0.01) decreased sleep latency and increased sleep duration as compared to the caffeine alone group. This suggests that RBS and DOX both possess the ability to counteract the wake-promoting effect of caffeine.

### 3.3. RBS Promotes NREMS without Changing REMS

**Figure 4:**
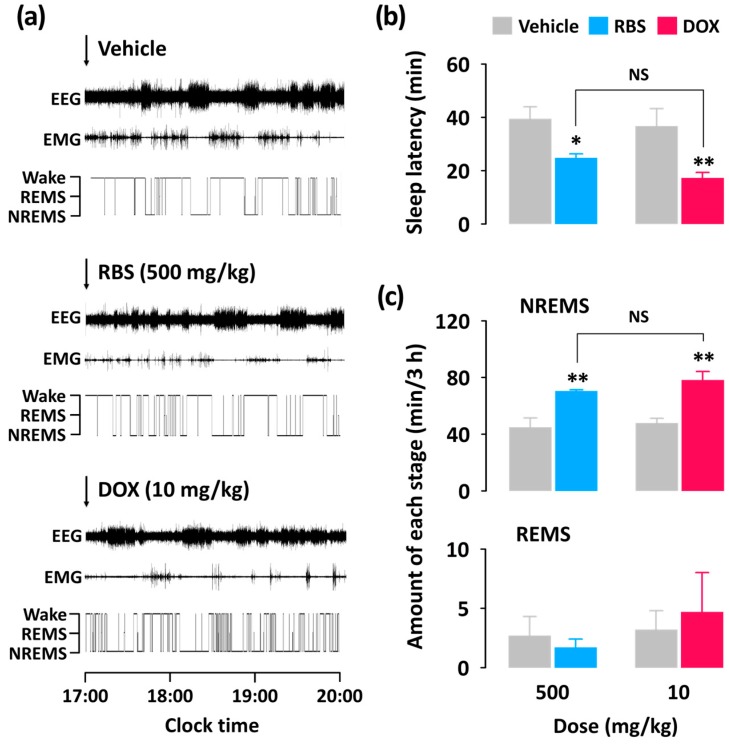
Effect of RBS and DOX on sleep-wake profiles in C57BL/6N mice. (**a**) Representative EEG and EMG signals, and corresponding hypnograms in a mouse treated with RBS or DOX; (**b**) Effects of RBS and DOX on sleep latency; (**c**) Amounts of NREMS and REMS during the 3 h period after administration of RBS or DOX. Grey bars indicate the baseline day (vehicle). Each value represents the mean ± SEM of each group (*n* = 7–8). * *p* < 0.05, ** *p* < 0.01, significantly different from vehicle (unpaired Student’s *t*-test). RBS, rice bran supplement; DOX, doxepin hydrochloride; EEG, electroencephalogram; EMG, electromyogram; Wake, wakefulness; REMS, rapid eye movement sleep; NREMS, non-REMS; NS, no significance.

**Figure 5:**
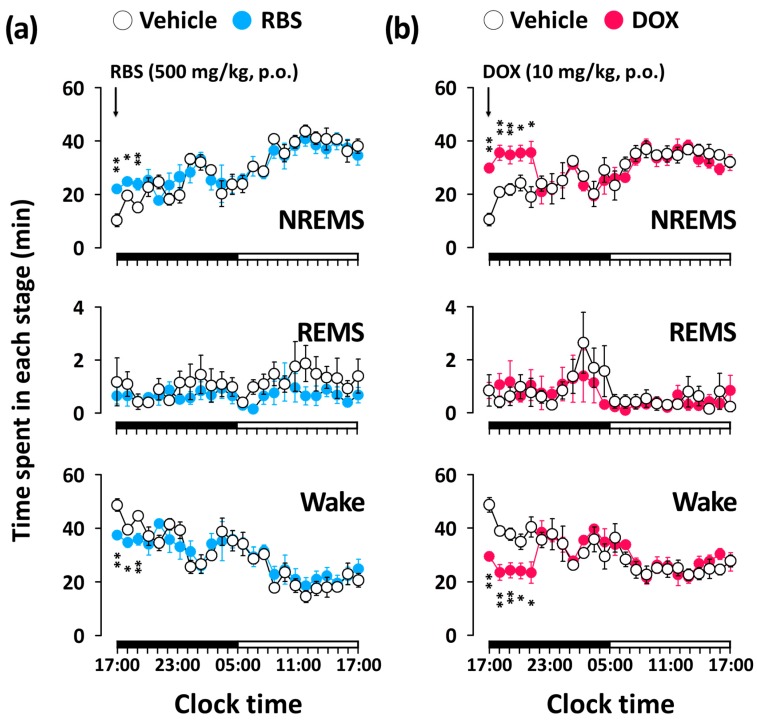
Effects of RBS (**a**) and DOX (**b**) on time-course changes in NREMS, REMS, and Wake during 24 h in C57BL/6N mice. Open and filled circles indicate the baseline day (vehicle) and experimental day (RBS or DOX), respectively. Each circle represents the hourly mean ± SEM (*n* = 7–8) of NREMS, REMS, and Wake. * *p* < 0.05, ** *p* < 0.01, significantly different from vehicle (unpaired Student’s *t*-test). The horizontal filled and open bars on the X-axis (clock time) indicate the 12-h dark and 12-h light periods, respectively. RBS, rice bran supplement; DOX, doxepin hydrochloride; Wake, wakefulness; REMS, rapid eye movement sleep; NREMS, non-REMS.

**Figure 6:**
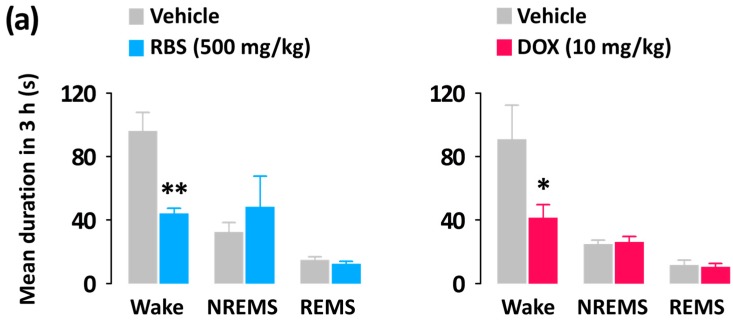

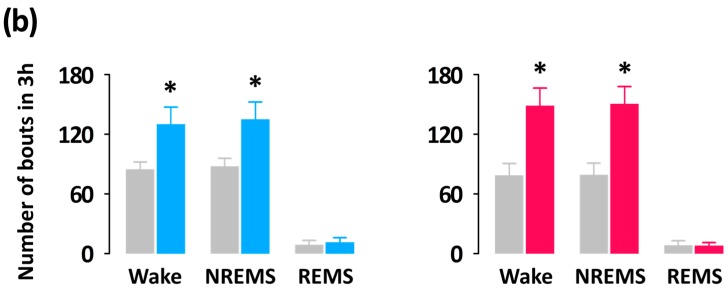
Characteristics of sleep-wake bouts in C57BL/6N mice during the 3 h period after administration of RBS and DOX. (**a**) Changes in the mean duration of Wake, NREMS, and REMS bouts; (**b**) Changes in the total number of Wake, NREMS, and REMS bouts. Grey bars indicate the baseline day (vehicle). Each value represents the mean ± SEM of each group (*n* = 7–8). * *p* < 0.05, ** *p* < 0.01, significantly different from vehicle (unpaired Student’s *t*-test). RBS, rice bran supplement; DOX, doxepin hydrochloride; Wake, wakefulness; REMS, rapid eye movement sleep; NREMS, non-REMS.

To better understand the hypnotic effect of RBS, we performed analyses of sleep architecture and sleep profiles in mice based on EEG and EMG recordings. Dosages of RBS and DOX were determined at 500 and 10 mg/kg, respectively, as these were the minimum dosages which showed statistical significance in the pentobarbital-induced sleep test (Figure 2). Figure 4a presents examples of EEG/EMG signals and corresponding hypnograms from a single mouse during the first 3 h following vehicle, RBS, or DOX administration. In the normal condition, without pentobarbital, RBS produced significant hypnotic effects. Administration of RBS (500 mg/kg) and DOX (10 mg/kg) decreased sleep latency (Figure 4b) by 1.59-fold and 2.13-fold, and increased NREMS by 1.57-fold, and 1.63-fold, respectively (Figure 4c). Moreover, RBS and DOX administration had no significant effect on REMS. Figure 5 shows the time course of changes in NREMS, REMS, and Wake during the 24 h period after RBS and DOX administration. RBS significantly increased the amount of NREMS recorded during the first 3 h of administration, and had no further effect on sleep architecture during the subsequent observation period (Figure 5a). On the other hand, the hypnotic agent, DOX, significantly increased the amount of NREMS recorded during the first 5 h of administration (Figure 5b). The mean duration of waking bouts in the RBS- and DOX-treated groups was significantly decreased during the first 3 h period after administration, but those of NREMS and REMS were not altered (Figure 6a). In contrast to the decrease in mean duration, RBS and DOX significantly increased the number of Wake and NREMS bouts (Figure 6b).

### 3.4. RBS May Exert Sleep-Promoting Effects via The Histaminergic Pathway

**Figure 7:**
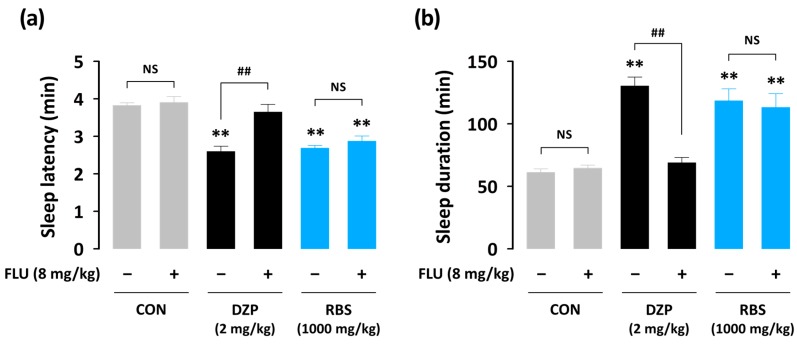
Effects of the GABA_A_-BZD receptor antagonist, FLU, on changes in sleep latency (**a**) and sleep duration (**b**) in ICR mice treated with DZP (2 mg/kg) and RBS (1000 mg/kg). Drugs were administered (p.o.) to mice 45 min before the injection of pentobarbital (45 mg/kg, i.p.). FLU (8 mg/kg, i.p.) was injected 10 min before the administration of test samples. Each column represents mean ± SEM (*n* = 10). ** *p* < 0.01, significant difference when compared to the CON group (Dunnett’s test). ## *p* < 0.01, significant difference between treatment with FLU and treatment without FLU (unpaired Student’s *t*-test). CON, control; DZP, diazepam; FLU, flumazenil; NS, not significant; RBS, rice bran supplement.

**Figure 8:**
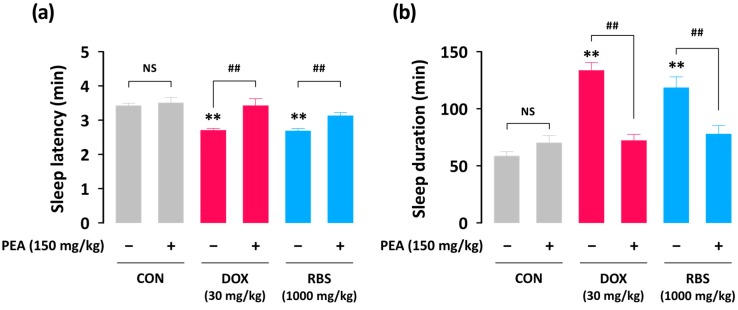
Effects of the H_1_R agonist, PEA, on changes in sleep latency (**a**) and sleep duration (**b**) in ICR mice treated with DOX (30 mg/kg) and RBS (1000 mg/kg). Drugs were administered (p.o.) to mice 45 min before the injection of pentobarbital (45 mg/kg, i.p.). PEA (150 mg/kg, i.p.) was injected 10 min before the administration of test samples. Each column represents mean ± SEM (*n* = 10). ** *p* < 0.01, significant difference when compared to the CON group (Dunnett’s test). ## *p* < 0.01, significant difference between treatment with PEA and treatment without PEA (unpaired Student’s *t*-test). CON, control; DOX, doxepin hydrochloride; NS, not significant; PEA, 2-pyridylethylamine dihydrochloride; RBS, rice bran supplement.

In order to explore the hypnotic mechanisms of RBS, we used drugs which could compete with GABA_A_-BZD receptor agonist and H_1_R antagonist binding. GABAergic and histaminergic systems are well-known molecular targets for sedative-hypnotic drugs [6,17]. FLU (a GABA_A_-BZD receptor antagonist) and PEA (an H_1_R agonist) were used as blockers to the GABA_A_-BZD receptor agonist, DZP, and the H_1_R antagonist, DOX, respectively. Pretreatment of FLU alone did not affect pentobarbital-induced sleep in mice (Figure 7). As expected, the GABA_A_-BZD receptor agonist, DZP (2 mg/kg), significantly decreased sleep latency and increased sleep duration–an effect that was fully blocked by administration of FLU (8 mg/kg). Unlike DZP, the hypnotic effect of RBS was not affected by FLU. In assessing the involvement of histamine signaling, we observed that the hypnotic effects of both DOX (30 mg/kg) and RBS (1000 mg/kg) were significantly inhibited by the H_1_R agonist, PEA (150 mg/kg) (Figure 8). These results suggest that the hypnotic effects exerted by RBS are likely mediated by the histaminergic, but not GABAergic, pathway.

## 4. Discussion

As a first step in evaluating the hypnotic activity of RBS, we performed a pentobarbital-induced sleep test on mice as this paradigm is often useful for evaluating sedative-hypnotic activity [18]. Our results clearly showed that, similar to an established insomnia medication, DOX, RBS significantly potentiated pentobarbital-induced sleep in mice (Figure 2). However, the potential of RBS as an effective natural sleep aid could not be solely determined using this primary result. Therefore, we further evaluated the sleep-promoting effects of RBS using a caffeine-induced sleep disturbance model, as well as an analysis of sleep structure and profiles based on EEG and EMG recordings.

When evaluating the sleep-promoting properties of a drug, it is important to note that results may not only reflect an agent’s hypnotic activity, but also its ability to counteract the impact of factors which disturb sleep [19]. Administration of caffeine, a well-known natural stimulant of the central nervous system, provides a simple and effective way to model one of the most common symptoms of insomnia [16,19]. The caffeine-induced sleep disturbance assay can be adopted both in animal and human studies, and has advantages, such as a simple protocol and low experimental cost [19]. Caffeine, an adenosine receptor antagonist, induces arousal through adenosine A_2A_ receptors [20], and reduces the build-up of occurrence of sleep pressure during wakefulness [21,22]. The arousing effect of caffeine on pentobarbital-induced sleep in mice has been observed in a previous study by Zhang et al. [23]. Thus, the fact that we observed a caffeine-induced increase in sleep latency and decrease in sleep duration compared to the control group was not entirely surprising (Figure 3). These arousal effects of caffeine in the pentobarbital-induced sleep test in mice were observed in previous study by Zhang et al. [23]. Co-administration of RBS with caffeine produced levels of sleep latency and duration that were similar to those observed in the control group. This result indicates that RBS may counteract the impact of caffeine on sleep disturbance. Considering global trends in coffee and energy beverage consumption, the attenuating effect of RBS on caffeine-mediated wakefulness adds to its value as a natural sleep aid.

Unlike the pentobarbital-induced sleep test, which addresses sleep latency and duration, analyses of sleep architecture and profile based on EEG and EMG recordings provide various parameters that are useful in evaluating the sleep-promoting effects of a drug [24]. Thus, in the present study, we evaluated the hypnotic effects of RBS on normal sleep in mice through evaluations of sleep architecture and sleep profile. Since normal sleep in rodents is physiologically fragmented, it could, by itself, represent a model of insomnia [19]. As nocturnal animals (e.g., rodents) rest during the day, evaluating the hypnotic effects of a drug in animals that are already prone to sleep during these hours may not be appropriate [25]. Thus, RBS and DOX were administered at 17:00, which corresponded to the beginning of the lights-off period. Using this approach, we found that both RBS and DOX significantly decreased sleep latency and increased the amount NREMS, but did not influence REMS (Figure 4). Moreover, the significant RBS-mediated increase in NREMS lasted for the first 3 h after administration, and had no further influence on sleep architecture (Figure 5). These results suggest that RBS was effective in increasing NREMS without causing adverse effects after sleep induction [26]. Finally, both RBS and DOX significantly decreased the mean duration of waking bouts, but increased the total number of waking and NREMS bouts (Figure 6). Together, these results clearly indicate that, similar to DOX, RBS was effective in inhibiting the maintenance of Wake [27].

Histamine, and its activity at H_1_ and/or H_3_ receptors, is known as a transmitter that is critical in the regulation of sleep-wakefulness. Previous studies have shown that wakefulness can be induced by histamine or H_1_ receptor agonist application, while H_1_ receptor antagonists have been shown to promote sleep [28,29]. In the current study, we revealed that the H_1_ antagonist-induced increase in pentobarbital-mediated sleep duration could be effectively inhibited by the H_1_ agonist, PEA. Interestingly, RBS, which increased sleep duration in a manner similar to DOX, was also inhibited by application of PEA (Figure 8). These results strongly suggest that RBS promotes sleep duration by acting as an H_1_ receptor antagonist.

γ-Oryzanol, which is a unique component of rice bran, has been widely known as the major active compound and the indicator constituent in the rice bran extracts or products [30]. RBS in this study was standardized to contain 4.5 mg/g of γ-oryzanol. Phytosterols, tocopherols, tocotrienols, and ferulic acid are known to be the major bioactive components of rice bran [13,31]. Among rice bran components, hypnotic effects of β-sitosterol and ferulic acid have been previously reported. Both β-sitosterol [32] and ferulic acid [33] potentiated the pentobarbital-induced sleep. Tu et al. [33] suggested that hypnotic effect of ferulic acid may be involved in the serotonergic system. According to our unpublished results, γ-oryzanol exhibits sleep-promoting effects through histamine receptors, like RBS. Based on these results, rice bran phytosterols, such as campesterol, stigmasterol, and β-sitosterol, may have potent hypnotic activity.

## 5. Conclusions

In the present study, we demonstrated the sleep-promoting effects of RBS and unveiled a possible mechanism of RBS action, implying a possible role for histaminergic signaling. Further studies addressing the precise mechanisms and active compounds responsible for mediating the hypnotic effects of RBS are needed. For example, our follow-up investigation is aimed at evaluating RBS binding affinity and antagonism at different histamine receptor subtypes (i.e., H_1_–H_4_). Together, our results suggest that RBS may be a promising natural sleep aid for treating insomnia or relieving mild sleep problems. In addition, our findings contribute to the growing body of evidence which support rice bran as a functional food and nutraceutical.

## Figures and Tables

**Table 1:** Proximate composition of the rice bran supplement (RBS).

Items	Content
Carbohydrate	46.3%
Moisture	6.6%
Protein	16.9%
Lipid	19.3%
Ash	10.9%
